# Sex differences in personality dysfunction in help-seeking adolescents

**DOI:** 10.1186/s40479-025-00287-2

**Published:** 2025-03-24

**Authors:** Marialuisa Cavelti, Jana Schenk, Silvano Sele, Corinna Reichl, Julian Koenig, Ines Mürner-Lavanchy, Michael Kaess

**Affiliations:** 1 University Hospital of Child and Adolescent Psychiatry and Psychotherapy, University of Bern, Bolligenstrasse 111, Bern 60, 3000 Switzerland; 2 Department of Child and Adolescent Psychiatry, Psychosomatics and Psychotherapy, Faculty of Medicine and University Hospital Cologne, University of Cologne, Cologne, Germany; 3 Faculty of Psychology, University of Basel, Basel, Switzerland; 4 Department of Child and Adolescent Psychiatry, Centre for Psychosocial Medicine, University Hospital Heidelberg, Heidelberg, Germany

**Keywords:** Personality disorder, Dimensional, DSM-5 Alternative Model of Personality Disorders (AMPD), Sex, Gender

## Abstract

**Introduction:**

Understanding sex differences is crucial for improving diagnosis and treatment for personality disorders (PDs). This study aimed to investigate sex differences in personality dysfunction as per Criterion A of the DSM-5 Alternative Model of Personality Disorders in help-seeking adolescents.

**Methods:**

The sample comprised 706 adolescent patients (mean age = 15.4 years; 80.88% females). Personality dysfunction was assessed using the Semi-Structured Interview for Personality Functioning DSM-5 (STiP 5.1).

**Results:**

Females showed significantly higher overall personality dysfunction (Cohen’s *d* = 0.36) compared to males, particularly in the self-functioning domain (*d* = 0.50), including identity (*d* = 0.52) and self-direction (d = 0.38). Sex differences in interpersonal functioning, particularly empathy, were statistically not significant, but females demonstrated greater impairments in intimacy compared to males (*d* = 0.23). Age did not moderate sex differences in personality dysfunction. Higher levels of personality dysfunction were associated with an increased likelihood of an alcohol use disorder and more severe psychosocial impairments in females compared to males.

**Discussion:**

The findings indicate that female adolescent patients exhibit greater impairments in personality functioning than males, with the difference being more pronounced in self-functioning than in interpersonal functioning. Results highlight the need for further investigation of the biological, psychological, and social factors driving these differences and call for the development of sex-sensitive diagnostic and interventional approaches to PDs.

**Supplementary Information:**

The online version contains supplementary material available at 10.1186/s40479-025-00287-2.

## Introduction

Personality disorders (PDs) are complex mental health conditions characterized by enduring patterns of behavior, cognition, and emotional functioning that deviate markedly from cultural norms [1]. They usually emerge during adolescence and young adulthood, impacting interpersonal relationships, education/work, physical health, and overall quality of life in the long term [2]. Notably, individuals with PDs face a heightened risk of suicide compared to those with no or other mental disorders [3]. This underscores the importance of prevention and early intervention for PDs in young people [4].

Historically, there has been reluctance to diagnose PDs, especially Borderline Personality Disorder (BPD), in adolescents, leading to delayed treatment [5]. However, recent evidence supports the reliability and validity of PD diagnoses in young individuals [6, 7]. The traditional categorical approach to PD diagnosis, criticized for its arbitrary thresholds, high comorbidity between different PD types, frequent use of “PD not otherwise specified” due to inadequate category fit, notable heterogeneity within the same PD type, as well as the inability to gauge the severity of PD pathology [8], has paved the way for the dimensional Alternative Model for Personality Disorders (AMPD) in Section III of the DSM-5 [1]). It assesses PD through the degree of impairment in self and interpersonal functioning (i.e., Criterion A) in combination with maladaptive personality traits (i.e., Criterion B), which are conceptualized as pathological versions of the Big Five dimensions. This dual-criterion approach to PD has also been adopted by the ICD-11, although determining specific personality traits (as suggested in Criterion B) is optional. Self and interpersonal functioning deficits are core features of PDs, distinguishing those with PD from those without PD and from those with other forms of psychopathologies, underlining our focus on Criterion A [9–11]. In addition, Criterion A predicts adverse outcomes, psychosocial adjustment, extending beyond categorical PD diagnoses [12–16], explains comorbidity among PD diagnoses [17], and is responsive to change [18]. Furthermore, it has been argued that Criterion A (but no Criterion B) accounts for the onset of PDs in adolescence. This is because abilities that fall within the purview of identity, self-direction, empathy, and intimacy (i.e., Criterion A) must be developed during this critical developmental period to enable adolescents to assume adult rights, responsibilities, and social and occupational roles [19, 20].

Prevalence, pathophysiology, and clinical manifestation of PDs as well as help-seeking behavior and treatment response can be influenced by differences between females and males [21, 22]. Sex is a genetic modifier of disease pathophysiology, clinical presentation, and treatment response, while gender can be considered a social and psychological modifier of disease presentation and of how, when, and why a person accesses professional care [23]. It is crucial to better understand sex and gender differences in PDs for tailoring prevention, diagnosis, and treatment strategies to enhance diagnostic precision and treatment effectiveness. However, research on sex/gender differences in PDs and sex-/gender-specific mental health care is still scarce. Existing research has concentrated on BPD and antisocial personality disorder (ASPD), noting variations in their prevalence and presentation between sexes [24]. BPD, historically regarded as predominantly affecting females [1], may have skewed gender ratios due to higher rates of treatment-seeking among women, with more balanced ratios observed in non-clinical samples [25, 26]. Males with BPD typically display externalizing symptoms (e.g., aggressiveness, impulsivity) and co-occurring disorders (e.g., substance use), while females with BPD exhibit internalizing symptoms (e.g., affective instability, self-harm) and co-occurring disorders (e.g., affective, anxiety, eating disorders) [27]. Men with BPD are also less likely than women to receive psychotherapy and psychiatric medication [28]. In ASPD, a higher prevalence is reported in men [24]. Women with ASPD show fewer episodes of antisocial behavior and a lower likelihood of cocaine use disorder, but higher rates of emotional and sexual abuse, and co-occurring borderline and histrionic PD, compared with men with ASPD [29]. Le Corff and colleagues [30] examined the prevalence rates for PD hybrid types derived from the AMPD. They observed higher prevalence rates (1) among younger adults compared to middle-aged and older adults, and (2) among men compared to women in the population sample. In contrast, the opposite was observed in the sample of young adults (aged between 17 and 22 years) at risk of developing PD due to a history of early conduct problems, where females exhibited higher prevalence rates than males. These findings align with epidemiological studies indicating that symptoms of (B)PD tend to peak during mid-to-late adolescence [31] before significantly attenuating over the course of adulthood [32]. The observed higher prevalence in young women of the at-risk sample may be attributed to the faster maturation of girls compared to boys [33], particularly during adolescence, which is also reflected in the development of psychopathology [34] and personality development [35]. Overall, these results highlight the critical role of age in understanding sex differences in personality pathology, particularly when examined during adolescence.

Sex/gender differences in other PDs, especially in non-adult populations, remain under-investigated [24]. Moreover, to the best of our knowledge, no studies have examined sex/gender differences in the extent of personality dysfunction (Criterion A) as defined by the DSM-5 AMPD in adolescents. This is notable, as Criterion A is fundamental to the diagnosis of all PDs, serving as the source rather than the consequence of maladaptive traits (Criterion B), and potentially accounting for the onset of PDs in adolescence [19, 36]. To address this gap, our study had the following aims: First, to examine sex differences in the prevalence of PD (categorical) and the degree of personality functioning impairments (dimensional) as per Criterion A of the AMPD in a clinical adolescent population. Second, to explore whether potential sex differences in personality dysfunction (dimensional) are dependent on the age at assessment. Third, to investigate whether sex moderates the associations between personality dysfunction (dimensional) and psychiatric comorbidity or impairments in psychosocial functioning. Throughout the study, we focused exclusively on sex assigned at birth, distinct from gender. By doing so, the study seeks to enhance the understanding of sex differences in personality dysfunction during adolescence, potentially informing sex-sensitive approaches to the assessment and treatment of PD pathology.

## Methods

### Participants and procedure

The sample for the present analysis consists of 706 help-seeking participants, pooled from two clinical cohort studies conducted at the University Hospital of Child and Adolescent Psychiatry and Psychotherapy in Bern, Switzerland, from November 2018 to March 2022. Participants were consecutively recruited from a specialized outpatient service for early detection and intervention for adolescents with PDs (AtR!Sk sample), and from general psychiatric day-care or inpatient services (Bernese Basic Documentation; BeBaDoc sample).

Inclusion criteria were age between 12–17 years (AtR!Sk sample) or 11–18 years (BeBaDoc sample), respectively, sufficient German language skills, and risk-taking or self-harming behavior (AtR!Sk sample). Participants who were not able to understand the study procedure and, therefore, were not able to give informed consent were excluded from the study. Participants over the age of 14 and caregivers (i.e., a parent or legal guardian) of participants under the age of 14 were required to give written informed consent. Data was collected using semi-structured interviews conducted by specifically trained clinicians (AtR!Sk study) or doctoral and psychology students (BeBaDoc study). Within the AtR!Sk study, assessments were integrated into the standard diagnostic evaluation process upon clinic admission, and, as a result, participants did not receive any compensation. In the BeBaDoc study, participants were provided with vouchers equivalent to a value of 20 CHF. The study protocols are in line with the declaration of Helsinki, and were approved by the local ethics committee (AtR!Sk Ethics ID: 2018 − 00942; and BeBaDoc Ethics ID: 2018 − 01339).

### Measures

Sociodemographic information such as age, biological sex based on self-report, level of education, and living situation were assessed using standardized interview questions. Diagnostic information was obtained with a short semi-structured clinical interview, the *Mini-International Neuropsychiatric Interview for Children and Adolescents (MINI-KID)* [37], which allows for the assessment of psychiatric disorders according to DSM-5 and ICD-10 [37]. The MINI-KID has been tested in psychometric evaluations as a valid and effective assessment instrument for this age group [38–40]. Further, BPD criteria were assessed using the *Structured Clinical Interview for DSM-IV axis II Personality Disorders (SCID-II-PD)* [41], which reflects categorical diagnostic criteria of BPD according to the DSM-IV that remained unchanged in DSM-5 Section II. The SCID-II-PD has been successfully applied to youth populations [42, 43]. The global functional capacity in daily life of the participants was assessed by two clinician-rated measures. The AtR!Sk study utilized the *Social and Occupational Functioning Assessment Scale (SOFAS)* [44], while the BeBaDoc study employed the *Children’s Global Assessment Scale (CGAS)* [45]. Both measures assess the patient’s overall level of functioning in social and occupational/educational areas, independent of the severity of psychopathology, and are rated on a scale ranging from 0 (minimum functional capacity) to 100 (maximum functional capacity).

The *Semi-Structured Interview for Personality Functioning DSM-5 (STiP 5.1)* was used to evaluate the severity of impairment in personality functioning, according to Criterion A of the AMPD [46]. Personality functioning encompasses the domains of self and interpersonal functioning, each comprising two elements with three facets, resulting in a total of four elements and twelve facets (see Supplementary Material (SM) Table 1). The degree of impairment for each facet is rated on a scale ranging from no/minimal impairment (0), mild (1), moderate (2), severe (3), to extreme (4), where higher scores indicate more severe impairment in personality functioning [1, 47]. For the current analysis, we employed the STiP 5.1 total score (i.e., mean value of the elements), domain scores (i.e., mean values of the corresponding elements), element scores (i.e., mean values of the corresponding facets), and facet scores. In addition, the diagnostic threshold for a PD diagnosis was defined as having a score of two or higher on two or more elements according to the AMPD guidance for trait-specified and specific PD [1]. The STiP 5.1 exhibits robust psychometric properties in an adult sample, including high internal consistency (Cronbach’s α for the total score: 0.97) and inter-rater reliability (interclass correlations (ICCs) ranging from 0.81 to 0.92) [46]. Likewise, it demonstrates good construct validity, and differentiates between clinical and non-clinical populations, as well as individuals with and without PD [46]. Notable, the STiP 5.1 has also shown commendable psychometric properties in an adolescent sample, with high internal consistency (Cronbach’s α for the total score: 0.96) and good interrater reliability (ICCs ranging between 0.88 and 0.99) [48].

### Statistical analyses

First, to examine sex differences in PD diagnosis according to the AMPD, a logistic regression was conducted with the STiP 5.1 PD diagnosis (yes/no) as the outcome variable and sex (0 = female, 1 = male) as the predictor. In addition, separate linear regression models were conducted to predict the degree of impairment in personality functioning (a) overall (i.e., STiP 5.1 total score), (b) on the level of domains, (c) the level of elements, and (d) the level of facets by sex. Age and dataset (AtR!Sk versus BeBaDoc) was included in all analyses as a covariate, as there is evidence for an increase in personality dysfunction from childhood to adolescence [31] and because it was expected that personality dysfunction is greater in the specialized outpatient service for early detection and intervention for PDs (i.e., AtR!Sk sample) compared to general psychiatric day-care or inpatient services (i.e., BeBaDoc sample).

Second, to examine whether the effect of sex was moderated by age, the linear regression models predicting personality dysfunction were repeated including the interaction term sex x age.

Third, to examine whether sex moderates the relationship between personality dysfunction and psychiatric comorbidity, logistic regression analyses were conducted using ICD-10 categories (F10-F90: yes/no) and the SCID-II BPD diagnosis (yes/no) as outcome variables. Although there is significant conceptual overlap between BPD and personality dysfunction [17], we treated BPD diagnosis as a psychiatric comorbidity in this analysis. This decision was based on findings from a recent study by our group, which unexpectedly identified a subgroup of adolescents with BPD, but without clinically significant personality dysfunction [49]. To explore if sex moderates the relationship between personality dysfunction and impairments in psychosocial functioning, linear regression analyses were performed with the SOFAS or CGAS, respectively, as the outcome variable. Sex, the STiP 5.1 total score, and the interaction sex x STiP 5.1 total score were included as predictors, while age and dataset (AtR!Sk versus BeBaDoc) were included as a covariate in all analyses.

Due to the exploratory nature of the analyses, we decided not to correct for multiple testing and to focus on effect sizes rather than significance levels when reporting and interpreting the results. Effect sizes were estimated using Cohen’s d, adjusted for age, with a value of 0.2 representing a small, a value of 0.5 representing a medium, and a value of 0.8 representing a large effect size. All statistical analyses were conducted using R (R Core Team, 2024) [50].

## Results

### Sample characteristics

From the total sample, 27 participants were excluded due to missing values in the STiP 5.1 total score (AtR!Sk sample: *n* = 4, BeBaDoc sample: *n* = 23). The final sample consisted of 706 adolescents, of which 448 (63.5%) received inpatient treatment (BeBaDoc study) and 258 (36.5%) received outpatient treatment (AtR!Sk). Sociodemographic and clinical characteristics for the total sample and the subsamples by sex are provided in Table 1. With 80.88%, the majority of adolescents were female. The mean age amounted to 15.4 (SD = 1.54) years. On average, adolescents had 3.02 (SD = 2.32) psychiatric diagnoses, with neurotic, stress-related, and somatoform disorders (ICD-F4), and affective disorders (ICD-F3) being the most common. The mean value of the total STiP 5.1 score was 1.14 (SD = 0.71), with higher values for impairments in the self functioning domain (M = 1.45, SD = 0.84) than the interpersonal functioning domain (M = 0.83, SD = 0.72). On average, only minimal to mild STiP 5.1 personality functioning impairments were observed, with mean values not reaching the clinical threshold of two. Descriptive statistics for the the AtR!Sk and BeBaDoc subsamples, disaggregated by sex, are presented in SM Table 2. In addition, descriptive statistics for the STiP 5.1 facets for the total sample and the subsamples by sex are provided in SM Table 3.

**Table 1:** Demographic and clinical characteristics of the total sample and the subsamples by sex

	Total sample		Females		Males	
	N (%)	M (SD)	N (%)	M (SD)	N (%)	M (SD)
Participants	706 (100)		571 (80.88)		135 (19.12)	
Age (in years)		15.4 (1.54)		15.4 (1.52)		15.6 (1.59)
Education						
Graduated from school (yes)	318 (45.0)		252 (44.1)		66 (48.9)	
Living situation						
Living with mother (yes)	603 (85.4)		485 (84.9)		118 (87.4)	
Missing	1 (0.1)		1 (0.2)		0 (0)	
Living with father (yes)	456 (64.6)		372 (65.1)		84 (62.2)	
Missing	10 (1.4)		6 (1.1)		4 (3.0)	
Treatment setting						
Inpatient / day care (yes)	448 (63.5)		381 (66.7)		67 (49.6)	
Outpatient (yes)	258 (36.5)		190 (33.3)		68 (50.4)	
STiP 5.1						
Total		1.14 (0.71)		1.18 (0.70)		0.98 (0.71)
Self functioning		1.45 (0.84)		1.52 (0.82)		1.17 (0.85)
Interpersonal functioning		0.83 (0.72)		0.84 (0.72)		0.80 (0.74)
Identity		1.66 (0.94)		1.74 (0.91)		1.34 (0.99)
Self-direction		1.25 (0.93)		1.30 (0.93)		1.00 (0.92)
Empathy		0.82 (0.76)		0.81 (0.75)		0.85 (0.81)
Intimacy		0.84 (0.86)		0.87 (0.85)		0.74 (0.88)
Diagnostic threshold (yes)	180 (25.5)		152 (26.6)		28 (20.7)	
SCID-II						
BPD (yes)	146 (20.7)		133 (23.3)		13 (9.6)	
Number of BPD criteria		2.75 (2.27)		2.99 (2.29)		1.76 (1.87)
MINI-KID						
Number of psychiatric diagnoses		3.02 (2.32)		3.22 (2.34)		2.21 (2.10)
ICD-10 F1 substance use disorders (yes)	219 (31.0)		180 (31.5)		39 (28.9)	
Missing	1 (0.1)		1 (0.2)		0 (0)	
ICD-10 F2 schizophrenia, delusional disorder (yes)	88 (12.5)		75 (13.1)		13 (9.6)	
Missing	5 (0.7)		4 (0.7)		1 (0.7)	
ICD-10 F3 affective disorders (yes)	418 (59.2)		359 (62.9)		59 (43.7)	
Missing	4 (0.6)		3 (0.5)		1 (0.7)	
ICD-10 F4 neurotic, stress-related, somatoform disorders (yes)	463 (65.6)		401 (70.2)		62 (45.9)	
Missing	4 (0.6)		3 (0.5)		1 (0.7)	
ICD-10 F5 disorders associated with physical factor (yes)	102 (14.4)		93 (16.3)		9 (6.7)	
Missing	4 (0.6)		3 (0.5)		1 (0.7)	
ICD-10 F9 disorders with onset in childhood and adolescence (yes)	308 (43.6)		249 (43.6)		59 (43.7)	
Missing	4 (0.6)		3 (0.5)		1 (0.7)	
Psychosocial impairments^a^		61.6 (16.5)		61.8 (16.4)		61.0 (17.0)

*BPD* Borderline Personality Disorder, *CGAS* Children’s Global Assessment Scale, *MINI-KID* Mini-International Neuropsychiatric Interview for Children and Adolescents, *SCID-II* Structured Clinical Interview for DSM-IV Personality Disorder, *SOFAS* Social and Occupational Functioning Assessment Scale, *STiP 5.1* Semi-Structured Interview for Personality Functioning DSM-5

^a^SOFAS (AtR!Sk) and CGAS (BeBaDoc) scores

### Sex differences in personality functioning impairments (study aim 1)

**Fig. 1:**
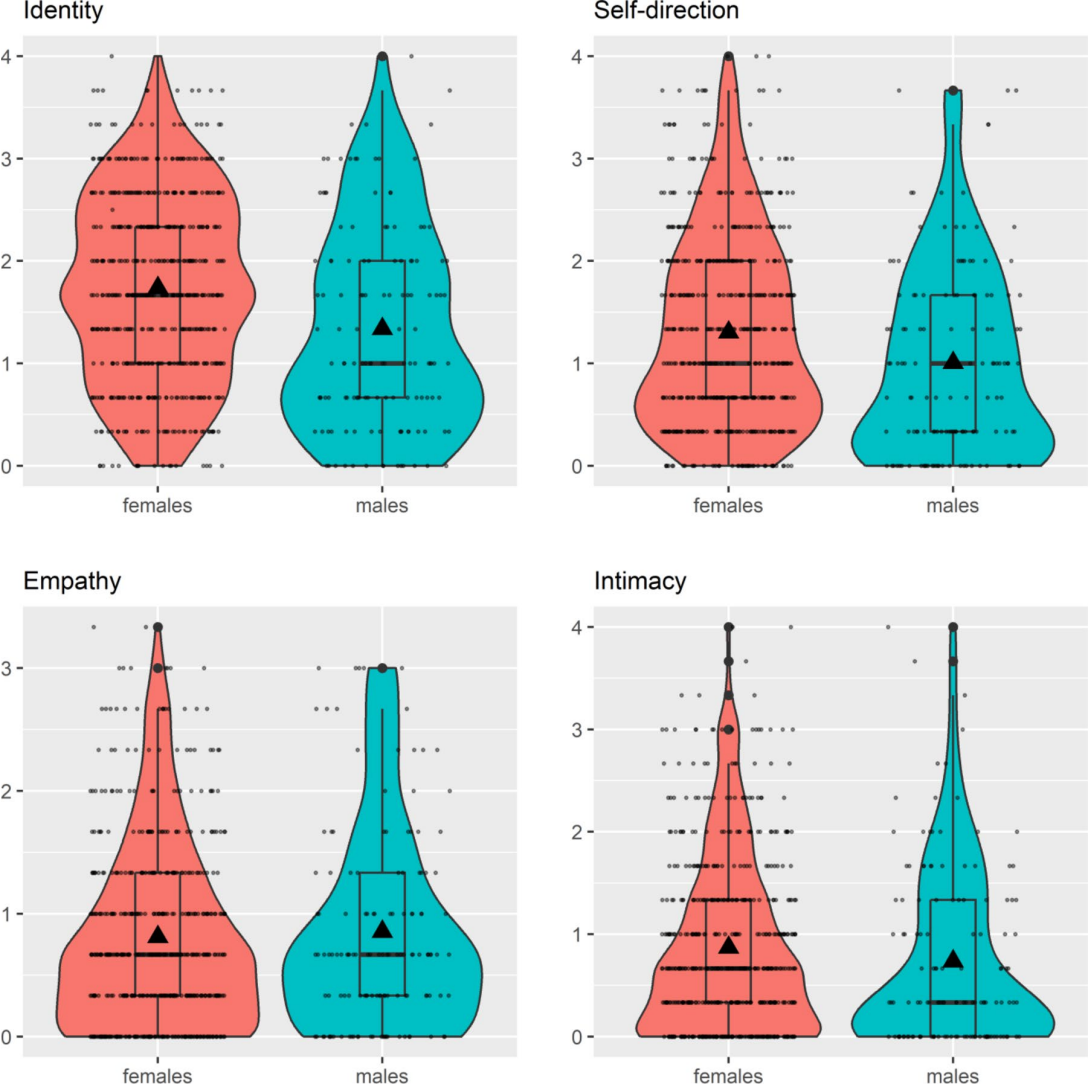
Scatter diagrams depicting the four STiP 5.1 elements identity, self-direction, empathy, and intimacy, grouped by sex and adjusted for age and dataset (AtR!Sk versus BeBaDoc). *Note.* Mean (▲) and median (**—**) values. The mean values are detailed in Table 2. STiP 5.1 = Semi-Structured Interview for Personality Functioning DSM-5

Females were significantly more likely to be diagnosed with PD according to the AMPD than males (OR = 0.63 [0.39, 0.99], *p* = 0.049)). In addition, as outlined in Table 2, females scored significantly higher on the STiP 5.1 total score than males (β = −0.25, 95% CI = [−0.38, −0.12], *p* = 0.0002), indicating greater overall impairment in personality functioning. Sex differences were particularly present in the self functioning domain (β = −0.41, 95% CI = [−0.56, −0.25], *p* = < 0.001), with the associated elements identity (β = −0.47, 95% CI = [−0.64, −0.30], *p* = < 0.0001) and self-direction (β = −0.35, 95% CI = [−0.52, −0.17], *p* =  0.0001). Cohen’s d ranged between 0.36 and 0.52, indicating small to medium effects. In contrast, there were non-significant sex differences in the interpersonal functioning domain and the associated element empathy, while females demonstrated greater impairments in intimacy compared to males, with a small effect size (β = −0.19, 95% CI = [−0.35, −0.03], *p* = 0.019, Cohen’s d = 0.23). A similar pattern was found on the level of facets (i.e., significantly higher levels of impairments in self functioning for females than males (small effects), with negligible sex differences in interpersonal functioning), with one exception: Females demonstrated significantly greater impairment in closeness than males, with a small effect size (β = −0.30, 95% CI = [−0.51, −0.10], *p* = 0.0043, d = 0.28). Full information on sex differences in the STiP 5.1 facets are given in SM Table 4. Sex differences in the elements are illustrated in Fig. 1; the illustration of the sex differences in the STiP 5.1 total, domain, and facet scores can be found in SM Figs. 1, 2 and 3.

**Table 2:** Sex differences in the STiP 5.1 total, domains, and elements

	Intercept females	Group difference males	Age	Dataset (AtR!Sk)	Cohens *d*
Total	1.10^***^ [1.04, 1.17]	−0.25^***^ [−0.38, −0.12]	0.05^**^ [0.01, 0.08]	0.24^***^ [0.13, 0.35]	0.36 [0.17, 0.55]
Self functioning	1.43^***^ [1.35, 1.51]	−0.41^***^ [−0.56, −0.25]	0.06^**^ [0.02, 0.10]	0.27^***^ [0.14, 0.39]	0.50 [0.31, 0.69]
Interpersonal functioning	0.77 ^***^ [0.70, 0.84]	−0.09 [−0.22, 0.05]	0.03 [0.00, 0.07]	0.21^***^ [0.10, 0.32]	0.12 [−0.07, 0.31]
Identity	1.64^***^ [1.56, 1.73]	−0.47^***^ [−0.64, −0.30]	0.09^***^ [0.05, 0.13]	0.29^***^ [0.15, 0.43]	0.52 [0.33, 0.71]
Self-direction	1.22^***^ [1.13, 1.31]	−0.35^***^ [−0.52, −0.17]	0.03 [−0.01, 0.07]	0.25^***^ [0.10, 0.39]	0.38 [0.19, 0.57]
Empathy	0.76^***^ [0.68, 0.83]	0.02 [−0.13, 0.16]	0.00 [−0.04, 0.04]	0.16^**^ [0.04, 0.28]	−0.02 [−0.21, 0.17]
Intimacy	0.79^***^ [0.71, 0.87]	−0.19^*^ [−0.35, −0.03]	0.06^**^ [0.02, 0.10]	0.25^***^ [0.12, 0.38]	0.23 [0.04, 0.42]

Adjusted for age and dataset (i.e., AtR!Sk versus BeBaDoc). Cohens d quantifies the group difference females– males divided by the between-person standard deviation. All values stated as mean [95% confidence interval]

*STiP 5.1* Semi-Structured Interview for Personality Functioning DSM-5

Significant at: ^*^p < 0.05, ^**^p < 0.01 and ^***^p < 0.001

### Moderation of sex differences in personality functioning impairments by age (study aim 2)

The interaction effect sex x age was not significant in any of the separate linear regressions for the STiP 5.1 total, domain, element, and facet scores, suggesting that the associations between sex and the STiP 5.1 scores were not moderated by age. However, significant effects of age on the STiP 5.1 total score (β = 0.05, 95% CI = [0.01, 0.08], *p* = 0.016), the self functioning domain (β = 0.06, 95% CI = [0.02, 0.10], *p* = 0.008), the elements identity (β = 0.09, 95% CI = [0.04, 0.14], *p* = 0.0002) and intimacy (β = 0.07, 95% CI = [0.02, 0.11], *p* = 0.0049), and the facets experience of oneself as unique (β = 0.08, 95% CI = [0.02, 0.14], *p* = 0.0083), self-esteem (β = 0.08, 95% CI = [0.02, 0.13], *p* = 0.0097), emotions (β = 0.12, 95% CI = [0.06, 0.18], *p* = < 0.0001), goals (β = 0.07, 95% CI = [0.00, 0.13], *p* = 0.047), connection (β = 0.06, 95% CI = [0.00, 0.12], *p* = 0.041), and closeness (β = 0.13, 95% CI = [0.07, 0.19], *p* = < 0.0001) were found, indicating greater impairments with increasing age. Full results of the models including the interaction term are given in SM Table 5.

### Sex differences in psychiatric comorbidity and impairments in psychosocial functioning associated with personality dysfunction (study aim 3)

A significant moderating effect of sex was found on the relationship between the STiP 5.1 total score and the likelihood of an ICD-10 F10 diagnosis (OR = 0.41 [0.22, 0.77], *p* = 0.0063). Specifically, for females, the likelihood of developing an alcohol use disorder was higher with higher levels of personality dysfunction (OR = 2.04 [1.55, 2.71], *p* < 0.0001), whereas this was not observed in males (OR = 0.85 [0.47, 1.48], *p* = 0.57). There was no moderating effect of sex on the relationship between the STiP 5.1 total score and the remaining ICD-10 categories (F20-F90). However, the wide confidence intervals suggest uncertainty in the estimates. In addition, a moderating effect of sex was identified on the relationship between the STiP 5.1 total score and psychosocial impairments (β = −3.44, 95% CI = [−6.84, −0.04], *p* = 0.047), with higher levels of personality dysfunction associated with greater psychosocial impairments in females than males. Again, the confidence interval was wide, indicating uncertainty. Full model results are provided in SM Table 6.

## Discussion

This is the first study to explore sex differences in the prevalence of PD (categorical) and the degree of personality functioning impairments (dimensional) according to Criterion A of the AMPD in a transdiagnostic sample of help-seeking adolescents. We found small to medium differences between females and males in personality functioning. Specifically, females were more likely than males to reach the diagnostic threshold for trait-specified and specific PD according to the AMPD and exhibited greater impairments particularly in self functioning. This was reflected in greater levels of impairments in identity, including the experience of oneself as unique, stable self-esteem and accurate self-appraisal, emotion regulation capacities, and self-direction, encompassing the ability to pursuit life goals, to utilize internal standards of behavior, and to reflect about oneself. Sex differences in interpersonal functioning, particularly empathy, were negligible. However, females showed slightly greater impairment in intimacy, particularly in their capacity for closeness than males (small effect size). Sex differences in personality functioning were not dependent on age at assessment. Additionally, increased levels of personality dysfunction were more strongly associated with the likelihood of alcohol use disorder and more severe psychosocial impairments in females than in males. Apart from this finding, there was little evidence of sex-specific associations between the level of personality dysfunction and the presence of ICD-10 mental disorders.

The observed sex differences align with previous research on categorical PDs indicating that in the clinical setting, PDs are more prevalent in women than in men [30]. In addition, the primary manifestation of sex differences in the self functioning domain is consistent with prior findings showing that: (a) women are more likely to be diagnosed with internalizing disorders (e.g., affective, anxiety, and eating disorders) compared to men who are more often diagnosed with externalizing disorders (e.g., attention-deficit/hyperactivity disorder, oppositional-defiant disorder, and conduct disorder) [51]; and (b) women with PD show more internalizing symptoms than men with PD [27, 29]. The observed sex differences in personality dysfunction may reflect the higher likelihood of females seeking professional help for mental health problems compared to males [52]. In addition, they may reflect diagnostic bias due to social and gender stereotypes [53, 54]. For instance, females are traditionally expected to display a keen interest in close relationships and exhibit a higher degree of prosocial behavior. Against this backdrop, even minor deviations from these normative expectations could lead to disproportionately higher scores in assessments of interpersonal functioning impairments for females, as demonstrated in the case of closeness in the current study. Furthermore, as Criterion A was developed based on research primarily conducted with individuals with BPD (the most extensively studied PD), who are predominantly female, there may be an inherent bias in the AMPD, which could potentially account for the observed sex differences in the current study. However, assuming that the sex differences in personality functioning impairment found in the current study reflect “true” disparities, the greater impairments observed in self functioning among females might be explained through an interaction of biological vulnerability (e.g., for increased emotional liability [55]) and environmental influences (e.g., sexual abuse [56], relational or social bullying experiences [57] or greater thinness-oriented body dissatisfaction due to social norms [58]) that may compromise identity formation during adolescence [59]. It could be hypothesized that the greater identity impairments among females contribute to the mental health crisis observed in female teenagers, which is characterized by an increase in depression, self-harm, and suicide attempts within this group [60–62].

Age did not influence the observed sex differences in personality dysfunction, but it did affect the severity of personality dysfunction across the total sample, suggesting that personality functioning impairments increase with age. This is in line with literature documenting a natural progression in PD features from early to mid- and late adolescence [31, 63].

The finding that the likelihood of an ICD-10 alcohol use disorder increases with higher levels of personality impairment in girls only is consistent with reports of a high co-occurrence between personality pathology, particularly BPD, and alcohol use in both adult and adolescent populations [64–66]. While problematic alcohol consumption is typically more common among male than female adolescents, the presence of personality pathology may diminish or even reverse this sex difference [67]. Alcohol use in individuals with personality pathology has been discussed as a maladaptive coping strategy to alleviate negative internal states and/or to fit in socially, as well as a means to seek sensation and enhance positive experiences [68, 69].

Finally, the sex-specific link between personality dysfunction and psychosocial impairments may suggest that personality dysfunction exerts greater impact on psychosocial functioning in females than males. However, this finding requires replication, given the considerable uncertainty in its estimate and its divergence from a previous study that reported no significant sex differences in psychosocial functioning among adults with BPD [70].

Strengths of the current study include the large sample of adolescent patients, and the assessment of the degree of personality dysfunction by a clinical interview conducted by trained researchers or clinicians, which is considered the gold standard in this diagnostic field. Several limitations are to be considered: First, the sex distribution in the sample was skewed towards females (i.e., 80.88% female), which is typical in clinical settings [71, 72] and aligns with other studies on youth with BPD [73, 74], but limits the generalizability of our findings and may have reduced the statistical power to detect sex differences in personality dysfunction. Second, the personality functioning impairments observed in the current sample were relatively mild in comparison to other adolescent patient samples [48], which could be explained by methodological differences. Weekers et al. [48] recruited patients from a mental health care center specialized in the assessment and treatment of PDs, where the majority of participants had full-threshold PDs. In contrast, our sample consisted of patients from two distinct settings: a specialized outpatient clinic for early detection and intervention of PDs, which includes a significant proportion of patients with sub-threshold PDs (i.e., the AtR!Sk sample), and general psychiatric day-care or inpatient services (i.e., the BeBaDoc sample), which are transdiagnostic by design. Additionally, differences in scoring systems between studies may account for variations in the reported levels of personality functioning. In our study, element scores were calculated as the means of their respective facet scores, domain scores were calculated as the means of their respective element scores, and the total score was computed as the mean of the domain scores. In contrast, Weekers et al. [48] utilized clinical judgment to derive scores across all hierarchical levels (facets, elements, domains, and total) (personal communication, January 8, 2025). Third, the study focused exclusively on sex as defined by the binary categorization of male or female at birth. Fourth, the study focused exclusively on Criterion A, neglecting Criterion B of the AMPD (i.e., maladaptive personality traits). Finally, we did not correct for multiple comparisons due to the exploratory nature of the study, which may have increased the risk for type 1 errors. Future research is warranted to explore sex differences in personality dysfunction in clinical samples with more balanced sex ratios as well as in non-clinical populations. Furthermore, it is important to explore how gender (in contrast to sex) influences personality functioning impairments. In addition, although diagnostic interviews are the gold standard for diagnosing PDs [6], future studies would benefit from adopting a multi-informant approach, combining clinician ratings with self and parent ratings. Moreover, further investigations into the biological, psychological, and social factors driving these sex differences are required. Finally, longitudinal studies are needed to investigate potential sex differences in the developmental trajectories of personality functioning impairments over time, as well as sex differences in the effectiveness of treatment approaches addressing personality dysfunction.

In conclusion, this study found that among patients aged 12–18 years with a range of mental disorders, females exhibited greater impairments in personality functioning than males. This difference was more pronounced in self functioning, particularly in identity, than in interpersonal functioning. These findings underscore the need for sex-sensitive treatment approaches. Females in particular may benefit from interventions that facilitate positive identity development (e.g., clarification of boundaries between self and others, boosting self-esteem and accurate self-appraisal, and improving emotion regulation abilities) for which interventions from Adolescent Identity Treatment (AIT) [75], Dialectical Behavioral Therapy for Adolescents (DBT-A) [76] or Mentalization-Based Treatment for Adolescents (MBT-A) [77] may be suitable. Furthermore, our findings highlight the importance of encouraging males to seek professional help. Strategies to achieve this may involve developing mental health care services that are more tailored to their specific needs, making them appear more appropriate and accessible to males.

## Supplementary Information

Supplementary Material 1.

## Data Availability

The datasets used and/or analyzed during the current study are available from the corresponding author on reasonable request.
