# Supplementary material for: Sex differences in personality dysfunction in help-seeking adolescents
Source: Borderline Personal Disord Emot Dysregul. 2025 Mar 24;12:10. doi: 10.1186/s40479-025-00287-2 (PMC11931781; doi:10.1186/s40479-025-00287-2)
Supplement: Supplementary file 1 — Supplementary Material 1. [file 40479_2025_287_MOESM1_ESM.docx]

**Supplementary Material for:**

**Sex differences in personality dysfunction in help-seeking adolescents**

Marialuisa Cavelti^1^, Jana Schenk^1^, Silvano Sele^1^, Corinna Reichl^1^, Julian Koenig^2^, Ines Mürner-Lavanchy^3^, Michael Kaess^1/4^

^1^ University Hospital of Child and Adolescent Psychiatry and Psychotherapy, University of Bern, Switzerland

^2^ University of Cologne, Faculty of Medicine and University Hospital Cologne, Department of Child and Adolescent Psychiatry, Psychosomatics and Psychotherapy, Cologne, Germany

^3^ Faculty of Psychology, University of Basel, Switzerland

^4^ Department of Child and Adolescent Psychiatry, Centre for Psychosocial Medicine, University Hospital Heidelberg, Germany

Includes:

- Table 1. Overview of domains, elements and facets as seen in the STiP 5.1
- Table 2. Demographic and clinical characteristics of the two subsamples (AtR!Sk verus BeBaDoc) by sex.
- Table 3. STiP 5.1 facets for the total sample and the subsamples by sex.
- Table 4. Sex differences in the STiP 5.1 facets.
- Table 5. Moderation of the sex differences in STiP 5.1 personality functioning impairments by age.
- Table 6. Sex differences in the relationship between STiP 5.1 personality dysfunction with psychiatric comorbidity or impairments in psychosocial functioning.
- Figure 1. Scatter diagram depicting the STiP 5.1 total, grouped by sex and adjusted for age.
- Figure 2. Scatter diagrams depicting the STiP 5.1 self- and interpersonal functioning domains, grouped by sex and adjusted for age.
- Figure 3. Scatter diagrams depicting the STiP 5.1 facets, grouped by sex and adjusted for age.

| ***Table 1.*** Overview of domains, elements and facets as seen in the STiP 5.1 [36]. | | |
| --- | --- | --- |
| Domains | Elements | Facets |
| Self-functioning | Identity | **Experience of oneself as unique**, with clear boundaries between self and others |
|  |  | **Self-esteem:** Stability of self-esteem and accuracy of self-appraisal |
|  |  | **Emotions:** Capacity for, and ability to regulate, a range of emotional experience |
|  | Self-direction | **Goals:** Pursuit of coherent and meaningful short-term and life goals |
|  |  | **Norms:** Utilization of constructive and prosocial internal standards of behavior |
|  |  | **Self-reflection:** Ability to self-reflect productively |
| Interpersonal functioning | Empathy | **Understanding others:** Comprehension and appreciation of others’ experiences and motivations |
|  |  | **Perspectives:** Tolerance of differing perspectives |
|  |  | **Impact:** Understanding the effects of one’s own behavior on others |
|  | Intimacy | **Connection:** Depth and duration of connection with others |
|  |  | **Closeness:** Desire and capacity for closeness |
|  |  | **Mutuality:** Mutuality of regard reflected in interpersonal behavior |
| *Note.* Facets are written in bold and described in regular font. STiP 5.1 = Semi-Structured Interview for Personality Functioning DSM-5. | | |

| ***Table 2.*** Demographic and clinical characteristics of the two subsamples (AtR!Sk versus BeBaDoc) by sex. | | | | | | | | | | |
| --- | --- | --- | --- | --- | --- | --- | --- | --- | --- | --- |
|  | AtR!Sk sample | | | | | BeBaDoc sample | | | | |
|  | Females | | | Males | | Females | | | Males | |
|  | N (%) | M (SD) | N (%) | | M (SD) | | N (%) | M (SD) | N (%) | M (SD) |
| Participants | 190 (33.3) |  | 68 (50.4) | |  | | 381 (66.7) |  | 67 (49.6) |  |
| Age (in years) |  | 15.3 (1.39) |  | | 15.3 (1.58) | |  | 15.4 (1.59) |  | 16.0 (1.54) |
| Education |  |  |  | |  | |  |  |  |  |
| Graduated from school (yes) | 91 (47.9) |  | 37 (54.4) | |  | | 161 (42.3) |  | 29 (43.3) |  |
| Living situation |  |  |  | |  | |  |  |  |  |
| Living with mother (yes) | 162 (85.3) |  | 63 (92.6) | |  | | 323 (84.8) |  | 55 (82.1) |  |
| Missing | 0 (0) |  | 0 (0) | |  | | 1 (0.3) |  | 0 (0) |  |
| Living with father (yes) | 127 (66.8) |  | 44 (64.7) | |  | | 245 (64.3) |  | 40 (59.7) |  |
| Missing | 0 (0) |  | 0 (0) | |  | | 6 (1.6) |  | 4 (6.0) |  |
| STiP 5.1 |  |  |  | |  | |  |  |  |  |
| Total |  | 1.34 (0.78) |  | | 1.08 (0.78) | |  | 1.10 (0.64) |  | 0.88 (0.62) |
| Self-functioning |  | 1.70 (0.89) |  | | 1.26 (0.93) | |  | 1.43 (0.77) |  | 1.08 (0.77) |
| Interpersonal functioning |  | 0.97 (0.82) |  | | 0.91 (0.84) | |  | 0.78 (0.65) |  | 0.68 (0.61) |
| Identity |  | 1.93 (1.01) |  | | 1.42 (1.09) | |  | 1.64 (0.84) |  | 1.25 (0.87) |
| Self-direction |  | 1.47 (0.95) |  | | 1.10 (0.96) | |  | 1.22 (0.90) |  | 0.91 (0.89) |
| Empathy |  | 0.91 (0.83) |  | | 0.96 (0.92) | |  | 0.76 (0.70) |  | 0.75 (0.68) |
| Intimacy |  | 1.03 (0.97) |  | | 0.85 (1.00) | |  | 0.79 (0.77) |  | 0.62 (0.71) |
| Diagnostic threshold (yes) | 69 (36.3) |  | 17 (25.0) | |  | | 83 (21.8) |  | 11 (16.4) |  |
| SCID-II |  |  |  | |  | |  |  |  |  |
| BPD (yes) | 52 (27.4) |  | 6 (8.8) | |  | | 81 (21.3) |  | 7 (10.4) |  |
| Number of BPD criteria |  | 3.26 (2.48) |  | | 1.75 (1.86) | |  | 2.85 (2.19) |  | 1.76 (1.90) |
| MINI-KID |  |  |  | |  | |  |  |  |  |
| Number of psychiatric diagnoses |  | 3.63 (2.48) |  | | 2.43 (2.27) | |  | 3.01 (2.24) |  | 1.99 (1.90) |
| ICD-10 F1 substance use disorders (yes) | 54 (28.4) |  | 16 (23.5) | |  | | 126 (33.1) |  | 23 (34.3) |  |
| Missing | 0 (0) |  | 0 (0) | |  | | 1 (0.3) |  | 0 (0) |  |
| ICD-10 F2 schizophrenia, delusional disorder (yes) | 25 (13.2) |  | 6 (8.8) | |  | | 50 (13.1) |  | 7 (10.4) |  |
| Missing | 2 (1.1) |  | 1 (1.5) | |  | | 2 (0.5) |  | 0 (0) |  |
| ICD-10 F3 affective disorders (yes) | 117 (61.6) |  | 26 (38.2) | |  | | 242 (63.5) |  | 33 (49.3) |  |
| Missing | 2 (1.1) |  | 1 (1.5) | |  | | 1 (0.3) |  | 0 (0) |  |
| ICD-10 F4 neurotic, stress-related, somatoform disorders (yes) | 147 (77.4) |  | 39 (57.4) | |  | | 254 (66.7) |  | 23 (34.3) |  |
| Missing | 2 (1.1) |  | 1 (1.5) | |  | | 1 (0.3) |  | 0 (0) |  |
| ICD-10 F5 disorders associated with physical factor (yes) | 50 (26.3) |  | 3 (4.4) | |  | | 43 (11.3) |  | 6 (9.0) |  |
| Missing | 2 (1.1) |  | 1 (1.5) | |  | | 1 (0.3) |  | 0 (0) |  |
| ICD-10 F9 disorders with onset in childhood and adolescence (yes) | 68 (35.8) |  | 32 (47.1) | |  | | 181 (47.5) |  | 27 (40.3) |  |
| Missing | 2 (1.1) |  | 1 (1.5) | |  | | 1 (0.3) |  | 0 (0) |  |
| Psychosocial impairments^a^ |  | 50.0 (13.90) |  | | 52.80 (15.90) | |  | 67.70 (14.20) |  | 69.30 (13.70) |
| *Notes.* BPD = Borderline Personality Disorder. CGAS = Children’s Global Assessment Scale. MINI-KID = Mini-International Neuropsychiatric Interview for Children and Adolescents. SCID-II = Structured Clinical Interview for DSM-IV Personality Disorder. SOFAS = Social and Occupational Functioning Assessment Scale. STiP 5.1 = Semi-Structured Interview for Personality Functioning DSM-5.  ^a^ SOFAS (AtR!Sk) and CGAS (BeBaDok) scores. | | | | | | | | | | |

| ***Table 3.*** STiP 5.1 facets for the total sample and the subsamples by sex. | | | | | | |
| --- | --- | --- | --- | --- | --- | --- |
|  | Total sample | | Females | | Males | |
|  | N (%) | M (SD) | N (%) | M (SD) | N (%) | M (SD) |
| Experience of oneself as unique |  | 1.31 (1.12) |  | 1.38 (1.12) |  | 1.01 (1.07) |
| Missing | 1 (0.1) |  | 1 (0.2) |  | 0 (0) |  |
| Self-esteem |  | 1.78 (1.12) |  | 1.87 (1.10) |  | 1.42 (1.16) |
| Emotions |  | 1.89 (1.15) |  | 1.96 (1.11) |  | 1.58 (1.27) |
| Goals |  | 1.11 (1.23) |  | 1.14 (1.22) |  | 0.96 (1.25) |
| Norms |  | 1.01 (1.25) |  | 1.05 (1.26) |  | 0.84 (1.18) |
| Self-reflection |  | 1.62 (1.11) |  | 1.72 (1.07) |  | 1.21 (1.18) |
| Understanding others |  | 0.71 (1.01) |  | 0.69 (0.98) |  | 0.80 (1.15) |
| Perspectives |  | 0.69 (0.91) |  | 0.70 (0.91) |  | 0.67 (0.92) |
| Impact |  | 1.06 (0.99) |  | 1.05 (0.98) |  | 1.09 (1.04) |
| Connection |  | 0.95 (1.10) |  | 0.97 (1.09) |  | 0.86 (1.13) |
| Closeness |  | 0.97 (1.11) |  | 1.02 (1.12) |  | 0.77 (1.08) |
| Mutuality |  | 0.61 (0.93) |  | 0.62 (0.93) |  | 0.58 (0.91) |
| *Notes.* STiP 5.1 = Semi-Structured Interview for Personality Functioning DSM-5. | | | | | | |

| ***Table 4.*** Sex differences in the STiP 5.1 facets. | | | | | |
| --- | --- | --- | --- | --- | --- |
|  | Intercept females | Group difference males | Age | Dataset (AtR!Sk) | Cohens d |
| Experience of oneself as unique | 1.28 ***  [1.18, 1.39] | -0.44 ***  [-0.65, -0.23] | 0.06 *  [0.01, 0.12] | 0.32 ***  [0.14, 0.49] | 0.40  [0.21, 0.59] |
| Self-esteem | 1.69 ***  [1.59, 1.80] | -0.55 ***  [-0.76, -0.35] | 0.09 ***  [0.04, 0.14] | 0.53 ***  [0.36, 0.69] | 0.51  [0.32, 0.71] |
| Emotions | 1.95 ***  [1.85, 2.06] | -0.42 ***  [-0.63, -0.20] | 0.12 ***  [0.07, 0.18] | 0.03  [-0.14, 0.21] | 0.37  [0.18, 0.56] |
| Goals | 1.04 ***  [0.92, 1.16] | -0.25 *  [-0.48, -0.01] | 0.05  [-0.01, 0.11] | 0.32 ***  [0.13, 0.51] | 0.20  [0.01, 0.39] |
| Norms | 0.91 ***  [0.79, 1.03] | -0.27 *  [-0.51, -0.04] | 0.01  [-0.05, 0.07] | 0.41 ***  [0.22, 0.60] | 0.22  [0.03, 0.41] |
| Self-reflection | 1.72 ***  [1.61, 1.82] | -0.52 ***  [-0.73, -0.31] | 0.03  [-0.02, 0.09] | 0.01  [-0.16, 0.18] | 0.48  [0.28, 0.67] |
| Understanding others | 0.64 ***  [0.54, 0.73] | 0.09  [-0.11, 0.28] | 0.01  [-0.04, 0.06] | 0.15  [0.00, 0.31] | -0.08  [-0.27, 0.11] |
| Perspectives | 0.65 ***  [0.56, 0.74] | -0.04  [-0.22, 0.13] | -0.01  [-0.06, 0.03] | 0.14 *  [0.00, 0.29] | 0.05  [-0.14, 0.24] |
| Impact | 0.99 ***  [0.89, 1.08] | 0.00  [-0.18, 0.19] | 0.01  [-0.04, 0.05] | 0.19 *  [0.04, 0.34] | -0.00  [-0.19, 0.19] |
| Connection | 0.88 ***  [0.77, 0.98] | -0.17  [-0.38, 0.03] | 0.06 *  [0.01, 0.11] | 0.28 **  [0.11, 0.45] | 0.16  [-0.03, 0.35] |
| Closeness | 0.97 ***  [0.86, 1.08] | -0.30 **  [-0.51, -0.10] | 0.12 ***  [0.07, 0.18] | 0.16  [-0.01, 0.33] | 0.28  [0.09, 0.47] |
| Mutuality | 0.52 ***  [0.43, 0.61] | -0.10  [-0.27, 0.08] | 0.01  [-0.04, 0.05] | 0.31 ***  [0.17, 0.45] | 0.11  [-0.08, 0.30] |
| *Notes.* Adjusted for age and dataset (i.e., AtR!Sk versus BeBaDoc). Cohens d quantifies the group difference females – males divided by the between-person standard deviation. All values stated as mean [95% confidence interval]. Significant at: * p <0.05, ** p <0.01 and *** p <0.001. STiP 5.1 = Semi-Structured Interview for Personality Functioning DSM-5. | | | | | |

| ***Table 5.*** Moderation of the sex differences in STiP 5.1 personality functioning impairments by age. | | | | | |  |
| --- | --- | --- | --- | --- | --- | --- |
|  | Intercept females | Group difference males | Age | Interaction sex (males) x age | Dataset (AtR!Sk) | |
| STiP 5.1 total | 1.10 ***  [1.04, 1.17] | -0.25 ***  [-0.38, -0.12] | 0.05 *  [0.01, 0.08] | 0.00  [-0.08, 0.09] | 0.24 ***  [0.13, 0.35] | |
| Self-functioning | 1.43 ***  [1.35, 1.51] | -0.41 ***  [-0.57, -0.25] | 0.06 **  [0.02, 0.10] | 0.01  [-0.09, 0.10] | 0.27 ***  [0.14, 0.40] | |
| Interpersonal functioning | 0.77 ***  [0.70, 0.84] | -0.09  [-0.23, 0.05] | 0.03  [-0.01, 0.07] | 0.00  [-0.08, 0.09] | 0.21 ***  [0.10, 0.32] | |
| Identity | 1.64 ***  [1.56, 1.73] | -0.47 ***  [-0.64, -0.30] | 0.09 ***  [0.04, 0.14] | 0.00  [-0.11, 0.10] | 0.29 ***  [0.15, 0.43] | |
| Self-direction | 1.22 ***  [1.13, 1.31] | -0.35 ***  [-0.53, -0.17] | 0.03  [-0.02, 0.08] | 0.02  [-0.09, 0.13] | 0.25 ***  [0.10, 0.39] | |
| Empathy | 0.76 ***  [0.68, 0.83] | 0.01  [-0.13, 0.16] | 0.00  [-0.04, 0.04] | 0.01  [-0.08, 0.10] | 0.16 **  [0.05, 0.28] | |
| Intimacy | 0.79 ***  [0.71, 0.87] | -0.19 *  [-0.35, -0.03] | 0.07 **  [0.02, 0.11] | -0.01  [-0.11, 0.09] | 0.25 ***  [0.12, 0.38] | |
| Experience of oneself as unique | 1.28 ***  [1.18, 1.39] | -0.43 ***  [-0.64, -0.22] | 0.08 **  [0.02, 0.14] | -0.08  [-0.21, 0.06] | 0.31 ***  [0.14, 0.48] | |
| Self-esteem | 1.69 ***  [1.59, 1.80] | -0.56 ***  [-0.77, -0.36] | 0.08 **  [0.02, 0.13] | 0.06  [-0.07, 0.19] | 0.53 ***  [0.36, 0.70] | |
| Emotions | 1.95 ***  [1.85, 2.06] | -0.42 ***  [-0.63, -0.20] | 0.12 ***  [0.06, 0.18] | 0.00  [-0.13, 0.14] | 0.03  [-0.14, 0.21] | |
| Goals | 1.04 ***  [0.92, 1.16] | -0.23 *  [-0.47, 0.00] | 0.07 *  [0.00, 0.13] | -0.08  [-0.23, 0.06] | 0.31 **  [0.12, 0.50] | |
| Norms | 0.91 ***  [0.79, 1.03] | -0.28 *  [-0.52, -0.05] | 0.00  [-0.07, 0.07] | 0.04  [-0.11, 0.19] | 0.41 ***  [0.22, 0.61] | |
| Self-reflection | 1.71 ***  [1.61, 1.82] | -0.53 ***  [-0.74, -0.33] | 0.01  [-0.04, 0.07] | 0.09  [-0.04, 0.22] | 0.02  [-0.15, 0.19] | |
| Understanding others | 0.63 ***  [0.54, 0.73] | 0.08  [-0.12, 0.27] | 0.00  [-0.06, 0.05] | 0.06  [-0.06, 0.18] | 0.16 *  [0.00, 0.32] | |
| Perspectives | 0.65 ***  [0.56, 0.74] | -0.04  [-0.22, 0.13] | -0.01  [-0.06, 0.04] | -0.01  [-0.12, 0.10] | 0.14 *  [0.00, 0.28] | |
| Impact | 0.99 ***  [0.89, 1.08] | 0.01  [-0.18, 0.20] | 0.01  [-0.04, 0.06] | -0.02  [-0.14, 0.09] | 0.19 *  [0.03, 0.34] | |
| Connection | 0.88 ***  [0.77, 0.98] | -0.17  [-0.38, 0.04] | 0.06 *  [0.00, 0.12] | -0.01  [-0.14, 0.12] | 0.28 **  [0.11, 0.45] | |
| Closeness | 0.97 ***  [0.86, 1.08] | -0.30 **  [-0.51, -0.09] | 0.13 ***  [0.07, 0.19] | -0.03  [-0.17, 0.10] | 0.16  [-0.01, 0.33] | |
| Mutuality | 0.52 ***  [0.43, 0.61] | -0.10  [-0.28, 0.07] | 0.00  [-0.05, 0.05] | 0.02  [-0.09, 0.13] | 0.31 ***  [0.17, 0.45] | |
| *Notes.* Adjusted for sex and dataset (i.e., AtR!Sk versus BeBaDoc). All values stated as mean [95% confidence interval]. Significant at: * p <0.05, ** p <0.01 and *** p <0.001. STiP 5.1 = Semi-Structured Interview for Personality Functioning DSM-5. | | | | | |  |

| ***Table 6.*** Sex differences in the relationship between STiP 5.1 personality dysfunction with psychiatric comorbidity or impairments in psychosocial functioning. | | | | | | | | | | | | | | |
| --- | --- | --- | --- | --- | --- | --- | --- | --- | --- | --- | --- | --- | --- | --- |
|  | | Intercept females | | Diff males | | STiP total (females) | | Age | | Males x STiP total | | STiP total (males) | | Dataset  (AtR!Sk) |
| ICD-10 F1 substance use disorder (yes) | 0.44 *** [0.34, 0.55] | | 0.83  [0.52, 1.30] | | 2.04 ***  [1.55, 2.71] | | 1.66 *** [1.46, 1.90] | | 0.41 **  [0.22, 0.77] | | 0.85  [0.47, 1.48] | | 0.73  [0.50, 1.05] | |
| ICD-10 F2 schizophrenia, delusional disorder (yes) | 0.15 *** [0.11, 0.20] | | 0.84  [0.42, 1.55] | | 1.89 ***  [1.35, 2.65] | | 0.94  [0.81, 1.09] | | 0.92  [0.39, 2.10] | | 1.73  [0.79, 3.71] | | 0.81  [0.49, 1.32] | |
| ICD-10 F3 affective disorders (yes) | 2.09 *** [1.68, 2.61] | | 0.58 *  [0.37, 0.91] | | 3.34 ***  [2.46, 4.62] | | 1.05  [0.94, 1.17] | | 1.37  [0.70, 2.85] | | 4.59 ***  [2.54, 8.97] | | 0.66 *  [0.46, 0.94] | |
| ICD-10 F4 neurotic, stress-related, somatoform disorders (yes) | 2.20 *** [1.77, 2.76] | | 0.36 *** [0.23, 0.56] | | 2.80 ***  [2.03, 3.94] | | 1.07  [0.96, 1.19] | | 1.15  [0.60, 2.30] | | 3.22 ***  [1.84, 5.97] | | 1.76 **  [1.21, 2.57] | |
| ICD-10 F5 disorders associated with physical factor (yes) | 0.13 *** [0.10, 0.18] | | 0.31 **  [0.12, 0.66] | | 2.05 ***  [1.50, 2.81] | | 0.94  [0.81, 1.09] | | 1.64  [0.64, 4.64] | | 3.37 **  [1.38, 9.13] | | 1.99 **  [1.27, 3.11] | |
| ICD-10 F9 disorders with onset in childhood and adolescence (yes) | 0.89  [0.72, 1.09] | | 1.29  [0.86, 1.95] | | 2.42 ***  [1.86, 3.17] | | 1.03  [0.93, 1.14] | | 0.91  [0.51, 1.64] | | 2.20 **  [1.32, 3.77] | | 0.59 **  [0.42, 0.82] | |
| SCID-II BPD diagnosis (yes) | 0.20 *** [0.15, 0.27] | | 0.37 **  [0.17, 0.72] | | 4.72 ***  [3.37, 6.75] | | 1.52 *** [1.31, 1.78] | | 0.69  [0.29, 1.70] | | 3.23 **  [1.47, 7.58] | | 1.01  [0.64, 1.57] | |
| Psychosocial impairments^a^ | 67.26 *** [66.02, 68.51] | | -0.20  [-2.69, 2.29] | | -8.16 ***  [-9.68, -6.63] | | -0.45  [-1.07, 0.17] | | -3.44 *  [-6.84, -0.04] | | -11.60 ***  [-14.65, -8.54] | | -15.56 ***  [-17.58, -13.55] | |
| *Notes.* Adjusted for age and dataset (i.e., AtR!Sk versus BeBaDoc). All values stated as mean [95% confidence interval]. Significant at: * p <0.05, ** p <0.01 and *** p <0.001. BPD = Borderline Personality Disorder. CGAS = Children’s Global Assessment Scale. SOFAS = Social and Occupational Functioning Assessment Scale. SCID-II = Structured Clinical Interview for DSM-IV Personality Disorders. STiP 5.1 = Semi-Structured Interview for Personality Functioning DSM-5.  ^a^ SOFAS (AtR!Sk) and CGAS (BeBaDok) scores. | | | | | | | | | | | | | | |


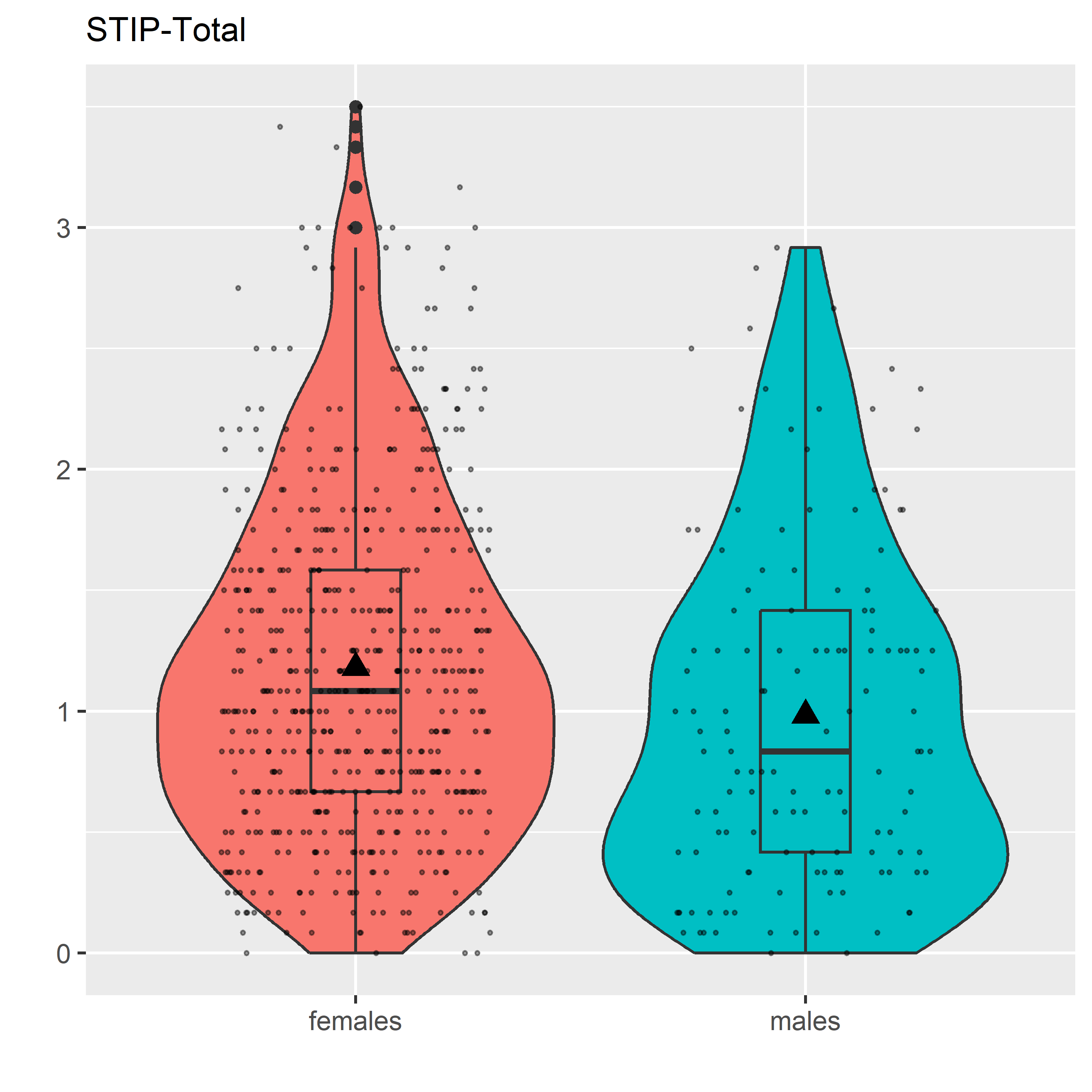


***Figure 1.*** Scatter diagram depicting the STiP 5.1 total, grouped by sex and adjusted for age and dataset (i.e., AtR!Sk versus BeBaDoc). *Note.* Mean (▲) and median (**—**) values. The mean values for the total scores are detailed in Table 2 in the main manuscript. STiP 5.1 = Semi-Structured Interview for Personality Functioning DSM-5.


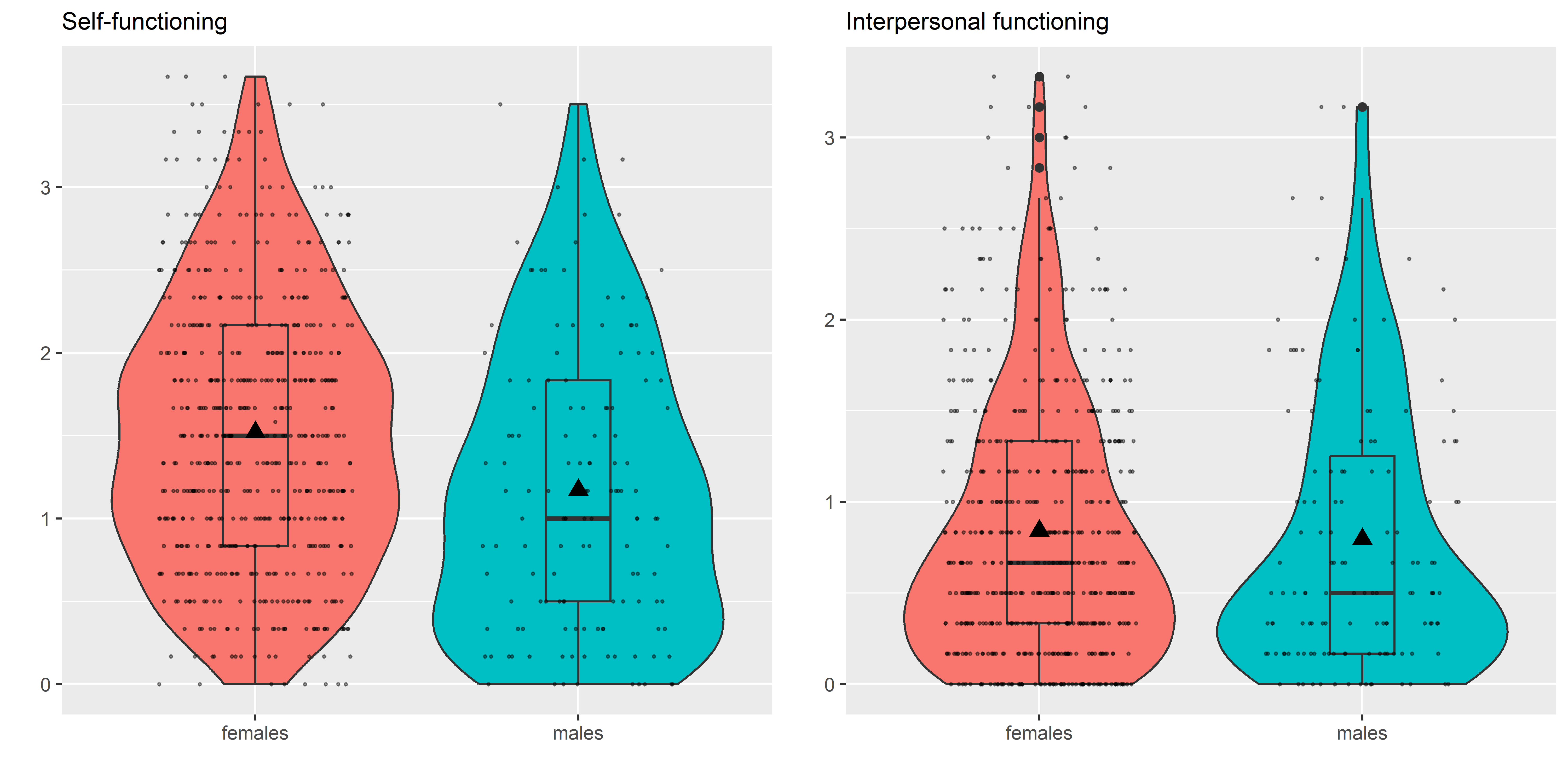


***Figure 2.*** Scatter diagrams depicting the STiP 5.1 self- and interpersonal functioning domains, grouped by sex and adjusted for age and dataset (i.e., AtR!Sk versus BeBaDoc). *Note.* Mean (▲) and median (**—**) values. The mean values for domain scores are detailed in Table 2 in the main manuscript. STiP 5.1 = Semi-Structured Interview for Personality Functioning DSM-5.





***Figure 3.*** Scatter diagrams depicting the STiP 5.1 facets, grouped by sex and adjusted for age and dataset (i.e., AtR!Sk versus BeBaDoc).. *Note.* Mean (▲) and median (**—**) values. The mean values for the facets are detailed in SM Table 2. STiP 5.1 = Semi-Structured Interview for Personality Functioning DSM-5.
